# Young adult perspectives on media content related to suicide in South India: a qualitative study

**DOI:** 10.1136/bmjopen-2025-113009

**Published:** 2026-05-27

**Authors:** Gregory Armstrong, Shuba Kumar, Rani Mohanraj, Sadhvi Krishnamoorthy, Lakshmi Vijayakumar

**Affiliations:** 1 Centre for Mental Health and Community Wellbeing, The University of Melbourne School of Population and Global Health, Melbourne, Victoria, Australia; 2 Samarth, Chennai, Tamil Nadu, India; 3 Department of Psychiatry, Voluntary Health Services, Chennai, Tamil Nadu, India; 4 Sneha Suicide Prevention Centre, Chennai, Tamil Nadu, India

**Keywords:** India, Suicide & self-harm, QUALITATIVE RESEARCH

## Abstract

**Objectives:**

The important role of responsible media reporting as a low-cost, effective population-level suicide prevention strategy is well documented. However, research into its potential to generate protective effects and how this is perceived by audiences is underexplored in the Indian context. This qualitative study aimed to explore young adults’ experiences of exposure to current suicide-related and purpose-designed content in the media.

**Design:**

This qualitative study was nested within a larger randomised controlled trial. A semi-structured interview guide was designed to explore participant perspectives and experiences regarding exposure to media content.

**Setting:**

The study was conducted with media professionals in Chennai, India.

**Participants:**

A purposive sample of 20 young adults (10 males, 10 females) participated in the study. Interviews were audio-recorded and transcribed, and an inductive-deductive approach to thematic analysis was followed.

**Results:**

Participants shared a range of contrasting experiences related to suicide content in the media and their responses to purpose-designed media content. They reported typical characteristics of current media reporting of suicides involving sensationalism, exaggeration and simplistic assumptions and underscored its impact on mental health. The purpose-designed content elicited a sense of surprise among participants. It stimulated curiosity, improved understanding, challenged misconceptions and instilled hope. In contrast to existing media coverage, they viewed protective content as a valuable means of educating people about recovery and encouraging help-seeking.

**Conclusions:**

This study uncovers unique insights into how young adults in the Indian context perceive and experience suicide reporting in the media. Our audience research indicates that current reporting styles may have harmful effects, while a more hopeful, recovery-oriented approach could offer significant benefits. These insights can be used to support meaningful collaborations between stakeholders in our efforts to encourage safe and respectful reporting that meets audiences’ needs to be kept informed.

**Trial registration:**

CTRI/2022/09/045439.

**STRENGTHS AND LIMITATIONS OF THIS STUDY**

- 20 qualitative interviews generated rich data, detailing insights into audience understanding of suicide-related media content.
- The study explicitly elicited participants’ reflections on protective, purpose-designed media content, an area that has received limited attention in previous research.
- Participants were urban, Tamil-speaking young adults from Chennai, which may limit transferability.

## Background

 Suicide rates in India are among the highest in the world with the most recent suicide death rate estimates ranging between 18 and 21 deaths per 100 000 population (cf, the global average of 11/100 000). This equates to an estimated 230 000–250 000 suicide deaths annually, which is approximately 28% of global suicides.^1 2^ Young people are a particularly important and high-profile group at risk in India, with the suicide rate among youth aged 15–29 years roughly double the national average.^2^ A public health approach to suicide prevention has gained momentum in India over the past decade,^3^ and the first national suicide prevention strategy was recently developed.^4^ The strategy adopts a multisectoral approach and identifies the critical role of media in suicide prevention.

Responsible media reporting of suicides is an important population-level strategy.^5^,^7^ The impact of media reports on the risk of suicide in the population has been extensively documented.^8^,^10^ Terms such as, ‘suicide contagion’, ‘copycat behaviour’ and the ‘Werther effect’ refer to an increase in the number of suicides in the population following such media reports.^9^,^11^ The risk of imitation is exacerbated by sensational and graphic reporting practices, for example providing a detailed description of the suicide method, especially of a celebrity suicide.^9 10 12^ Furthermore, media reports can unintentionally lead to the dissemination of suicide methods and behaviours^13^ and can be a source of misinformation through providing simplistic monocausal explanations for suicide.

However, there has been limited research exploring the potential of media stimuli to generate protective effects. The few ecological studies that have examined this have observed that suicide rates reduce following non-fictional and fictional media stories about people who have found ways to cope through periods of suicidal thoughts.^9 14^ This has become known as ‘the Papageno effect’. A recent meta-analysis of eight experimental studies, all undertaken in either Austria or Australia, observed that exposure to educative suicide prevention media content containing stories of hope and recovery from a suicide crisis can have protective effects on suicidal ideation among people with greater pre-existing vulnerability.^15^

In 2023, the WHO and the International Association for Suicide Prevention partnered to develop international media guidelines on reporting of suicides for media professionals.^16^ These guidelines recommend that media coverage avoid potentially harmful practices, such as sensational reports of suicidal behaviour and providing detailed descriptions of suicide methods. The guidelines also recommend potentially helpful practices, such as educating the public about suicide, highlighting suicide prevention services and programmes and reporting on stories of people who have coped through a period of suicidal thoughts. In 2019, the Press Council of India issued context-specific guidance for India, drawing on earlier recommendations from the WHO media guidelines.^17^ The guidance encouraged media professionals to ensure accurate and fair reporting by verifying facts, protecting privacy and maintaining balanced coverage in the public interest. While this marks an important step toward addressing the issue, much of the existing evidence on media reporting is drawn from high-income countries. Our recent systematic review indicates that an evidence base is beginning to emerge in low- and middle-income countries (LMICs), but it remains concentrated in few countries and dominated by newspaper analyses.^18^

In India, our research has found that suicide deaths and attempts are covered frequently, graphically, explicitly and simplistically, predominantly by crime journalists.^19^,^21^ For example, newspapers publish on average one suicide article per day, typically brief (≤10 sentences) incident reports. Potentially harmful reporting practices such as detailing suicide methods are common while helpful practices, expert commentary or crisis support information, are rare. Hopeful survival stories are absent; only one was found among >1600 articles on suicide.^19^ Other studies from India have documented similar findings.^20^,^26^ Our previous qualitative study with media professionals in India revealed that suicides were seen as being highly newsworthy, and often used as ‘clickbait’, with a notable focus on youth suicides due to their sensational appeal.^27 28^ Therefore, adherence to media guidelines and its impact on reporting quality indicates inconsistent implementation across LMICs such as India.^20 21 29^

Evidence from India is needed to understand how improved media reporting can create protective effects at the population level. To address this gap, we conducted a multi-arm randomised controlled trial (RCT) with young adults in South India to evaluate the effects of exposure to educative suicide content and stories of surviving a suicide crisis. Young adults are an important suicide risk group in India, making them an important population for this study. Suicidal ideation was measured as the primary outcome. Secondary outcomes included young adults’ stigmatising attitudes, suicide prevention knowledge and intentions to seek help, assessed before and after exposure to the intervention and control conditions. Findings from the trial are being reported elsewhere.^30^ Alongside the trial we conducted a qualitative study involving participants in the trial who had been exposed to the purpose-designed protective media content. This qualitative study aimed to explore young adults’ experiences of exposure to suicide-related content in the media prior to the study, and their perspectives on the purpose-designed protective media content they were exposed to during the trial. To our knowledge, this study represents the first qualitative enquiry into the public’s experiences of consuming media coverage of suicide in India. The perspectives of the young adults in this trial will be important for informing initiatives to engage media professionals on their approach to covering suicide-related news.

## Method

### Study design

This qualitative study was nested within a larger RCT which examined the effects of exposure to educative suicide prevention content and stories of surviving a suicide crisis in the print media on reducing suicidality in India. A three-arm RCT was conducted in Chennai, India (August 2023–March 2024). The study involved a collaboration between researchers from Samarth, a non-governmental organisation engaged in social science research based in the city of Chennai, Tamil Nadu in South India and researchers from the Melbourne School of Population and Global Health at the University of Melbourne. Young adults aged 18–29 years were recruited through social media advertisements and randomised (1:1:1) into a control group and two intervention groups. All groups were exposed to three purpose-designed 600-word Tamil-language print articles on digital tablets. Participants were exposed to print media articles that included coverage of the new national suicide prevention strategy; examples of suicide prevention programmes; debunking suicide myths; opinions from mental health experts; encouraging people to seek help; and messages of hope that people with suicidal thoughts can find a way to survive a suicide crisis with support (intervention arm 1). For the second intervention group, the same articles were modified to also contain brief lived experience accounts of young adults who had coped with experiences of suicidal ideation. These examples highlighted help seeking behaviours, a range of different coping strategies and support mechanisms (intervention arm 2). The control group received three articles on diabetes.

The qualitative study aimed to examine young adults’ experiences of exposure to suicide-related content in the media prior to the study and their perspectives on the purpose-designed protective media content they were exposed to during the trial. Semi-structured interviews (SSIs) were used to explore participant experiences and perspectives.

The study was approved by the Human Research Ethics Committee at the University of Melbourne (Ref: 2021–20927-15388-3) and by the Ethics Committee of the Schizophrenia Research Foundation (SCARF) in India (Ref: SRF-SAMARTH/02/FEB-2022), a WHO Collaborating Centre for Mental Health Research and Training. The study also received approval from the Health Ministry Steering Committee at the Indian Council of Medical Research. The larger trial was registered with the Clinical Trials Registry of India (Ref: CTRI/2022/09/045439). Consolidated criteria for reporting qualitative research (COREQ; ^31^ online supplemental material 1) checklist was followed.

### Sample

Young adults aged 18–29 years of age living in Chennai who were literate in Tamil were recruited as part of the RCT. Participants recruited for the qualitative study were drawn from the two intervention groups who were exposed to educative suicide prevention print media content, as we were interested in their perspectives on this potentially helpful media content. Following completion of their participation in the trial, participants were approached to understand if they had time and willingness to participate in a semi-structured qualitative interview. Our aim was to recruit an equal representation of males and females (n=10 each) from both intervention groups. Those who agreed to participate were subsequently contacted. Non-response was not noted. Given our focused objectives and the homogeneity of the participant group (ie, Tamil-speaking urban youth), we had estimated 20 participants would be sufficient.^32 33^ Participants signed a written consent form before the commencement of the interviews.

### Data collection

The research team comprised investigators with diverse expertise in health and mental health research, including GA (Male, PhD in International Health), SK (Female, PhD in Social Psychiatry), RM (Female, PhD in Psychology), SKr (Female, PhD in Public Health) and LV (Female, PhD and MD in Psychiatry). Two senior researchers (SKu and RM) employed as a social scientist/researcher and a psychologist respectively, led the interview process. Both the interviewers had extensive training in quantitative and qualitative research methods and had participated in national and international multi-centric health projects. The remaining three team members (GA, SKr, LV) had extensive experience in mental health and suicide prevention research and implementation in both Australia and LMIC settings.

Participants were assigned a unique identification number during baseline data collection prior to the trial, when demographic information was collected. Those who took part in the qualitative study provided this ID at the start of the interview process, allowing the research team to retrieve their demographic data without requiring them to repeat this information. All interviews were conducted face-to-face, within 2 weeks of their participation in the trial. The interviews were conducted in Tamil, in a private office setting and were audio recorded. No one else was present in the room except the interviewers with their respective participants. Interviews lasted for 45–60 min and were undertaken between December 2023 and February 2024. Before the interview commenced, the interviewers introduced themselves. Participants were given an opportunity to understand what the interview process entailed. They were given time to re-read the print media content they had been given as part of the study to refresh their memory. Interviewers (SKu and RM) had no prior relationship with the participants. As part of introducing themselves, the interviewers shared their name, qualifications and professional experience with the participants. They shared the objectives of the study and reported their interest in understanding how audiences perceive suicide-related news in the media.

An interview guide (online supplemental material 2) was developed keeping in mind several objectives: (1) to explore their experiences and perceptions of suicide-related media content they typically encounter in the real world; (2) to examine their experiences and perspectives of the educative suicide prevention media content they were exposed to during the trial and how it differed from the media content they typically see. Additionally, the interviews aimed to explore the influence of the content on participants’ experiences of distress (if any; primary outcome), as well as their suicide prevention knowledge, stigmatising attitudes and help-seeking behaviours (secondary outcomes). Open-ended questions with probes were developed to address these objectives. After 20 interviews, the research team determined that a sufficient range of responses had been obtained, since no new issues emerged in the discussion. Recruitment was therefore ceased following discussion with senior members of the team (GA and LV). No repeat interviews were carried out.

### Researcher reflexivity

The research team comprised a mix of disciplines, including social science researchers and mental health clinicians (psychiatry, social work, psychology). Our perspective on this research was informed by a suicide prevention and public health lens, more than a media communications lens. Notes and memos capturing key interview findings were maintained throughout the process. Two researchers (SKu and RM) met regularly to discuss and reflect on insights obtained during the data collection process. Periodic zoom calls with another senior researcher (GA) facilitated further discussions on issues identified during the interviews, providing an independent perspective

### Data analysis

All the audio recorded SSIs were transcribed verbatim, translated into English, shared with the research team and loaded into NVivo. Due to time and resource constraints, transcripts were not returned to participants for comments and/or correction. Therefore, participants did not have time to provide feedback on the transcripts. The researchers actively interpreted the data through the coding process, developing code categories and subsequently identifying themes.^34^ Field notes taken after each interview and analytic memos were maintained throughout the coding process.

A deductive and inductive thematic analytic approach was used^35^ to corroborate and complement findings of the trial. An initial code list based largely around the topic areas discussed in the interview guide was first developed which was followed by a six-stage thematic analysis process.^35^ The first stage involved data immersion through repeated readings of interview transcripts. In the second stage, SKu and RM independently coded two transcripts using the initial code list developed *apriori*. The data sets of the two coders were merged and a codebook generated that was reviewed by all authors. Codes for the remaining transcripts were derived inductively, driven by the data. These new codes contributed to the identification of sub-themes under the pre-determined major themes. Similar codes were merged, and discrepancies were discussed and resolved. The finalised codebook was then used to code the remaining transcripts with new codes added when required as the analysis progressed. In the third stage, coded data were examined to form categories aligning with our research objectives. The fourth stage involved refining and validating themes for coherence and credibility. In the fifth stage, themes were labelled and defined and in the final stage study findings were synthesised, with representative quotes selected to illustrate each theme.

### Study rigour

Some steps were undertaken to ensure rigour in the study design and conduct.^36^ To ensure credibility of findings, the study was conducted by researchers based in Chennai who were well familiar with and understood the context. This also enabled an environment where trust could be built with participants in the interview process. Triangulation of quantitative and qualitative data was purposely designed to ensure a holistic understanding of study outcomes is achieved. In addition, multiple researchers were involved in the analysis process and regular peer debriefings were conducted to refine interpretations.

Transferability was addressed pragmatically. While a detailed thick description of the context could not be documented due to resource constraints, key contextual information about participants and the setting was used to aid interpretation. Dependability was supported through continuous discussions conducted with the principal investigator (GA) who reviewed key aspects of the research process and analytic decisions made. Finally, confirmability was strengthened through reflexivity among researchers and triangulation of data sources and analytic perspectives.

## Results

### Participant characteristics

The sample comprised an equal number of males (n=10; 50%) and females (n=10; 50%) aged between 18 and 29 (see table 1). Most participants were undergraduate or postgraduate students specialising in a range of disciplines such as science, commerce, fashion, creative arts and practice. Of these, eight participants were employed in various roles, including positions in an IT company, as a manager in a private hospital, as an athlete, in sales and as a photographer. Most participants (n=18, 90%) did not report current suicidal thoughts (in the past 12 months). Participants were also asked about their recent exposure to suicide-related news. While one participant could not recall any such exposure, 40% of the participants (n=8) indicated that they had not been exposed to such news at all in the past 2 weeks. A few (n=5, 25%) mentioned that they had been exposed to suicide-related news once or twice (n=6, 30%) in the past 2 weeks.

**Table 1:** Participant characteristics

Characteristics	N (%)
Gender	
Male	10 (50)
Female	10 (50)
Age	
18–23	14 (70)
24–29	6 (30)
Education	
High school	3 (15)
Tertiary education	17 (85)
Occupation	
Student	12 (60)
Employed	8 (40)
Suicidal thoughts (past 12 months)	
Yes	2 (10)
No	18 (90)
Recent exposure to suicide-related news (past 2 weeks)	
Never	8 (40)
Once	5 (25)
Twice or more	6 (30)
I don’t remember	1 (5)

Participants shared a range of experiences encapsulated in two major thematic areas based around issues explored in the interview guide: (1) exposure to media coverage of suicide in the past and (2) exposure to the purpose-designed media content on suicide with protective reporting features. There were six sub-themes within these major themes (see table 2).

**Table 2:** List of themes and sub-themes

Major themes	Definitions	Sub-themes
Exposure to media coverage of suicide	Participants’ experiences and perspectives of suicide in the media and its perceived impact on themselves and others	Chasing ratings: sensationalism, exaggeration and simplistic assumptions Effects of exposure: sadness, disturbance and avoidance Missed opportunities to educate the public on how to cope
Exposure to purpose-designed media content on suicide with protective reporting features	Participants’ experiences and perceptions of the educative and hope-oriented media content related to suicide provided to them in the study, and its perceived impact	Hope and recovery Sparking curiosity, improving knowledge, busting myths Sources of support

### Exposure to media coverage of suicide in the past

Participants described their experiences of the suicide-related news stories they had encountered prior to the trial. This has been discussed as three sub-themes.

#### Chasing ratings: sensationalism, exaggeration and simplistic assumptions

Participants generally understood that media houses, especially television channels, faced pressures to boost their ratings and used suicide news to attract their audience*—‘*They highlight the death of the person just for the sake of their [ratings]’ (Male #7). Referring to the level of audience interest, participants reflected that suicide-related news was often given prominent coverage and could be found ‘printed on the front page [of newspapers]*’* (Male #7). They also pointed to a bi-directional relationship, whereby media houses used sensational approaches to gain audience interest, while high audience interest in suicide news also drove the media to feed this interest. ‘People are very much interested in this… [the media] show it in the way people want it… and they sensationalise things for their ratings’ (Female #2).

As a result of the chase for ratings, participants felt that suicide-related news was characterised by ‘drama and exaggeration*’* (Female #3). Participants primarily described seeing incident-based news stories that sensationally documented suicide deaths and attempts in the community using eye-catching headlines, photos, and giving very graphic details about the suicide and the people involved. Participants expressed discomfort in being exposed to details of the person who had died. ‘We only get to see a graphic description [of suicide] now-a-days on TV… they make assumptions and exaggerate the content*’* (Male #8), and ‘they will post pictures, show blurred faces, some people may get disturbed*’* (Female #9).

Participants frequently raised doubts about the veracity and credibility of these media reports, viewing them as a means for media outlets to attract attention and widen readership/viewership. They believed that reports on suicide were often exaggerated in content with numerous assumptions made about the reasons for suicide and surrounding circumstances which did not necessarily reflect reality, ‘they assume the reasons for a suicide… they make so many assumptions*’* (Male #3).

#### Effects of exposure: sadness, disturbance and avoidance

Participants reported experiencing a range of emotions when encountering suicide-related news. They described feeling sad, distressed when reading about individuals who had attempted or died by suicide and continued to ruminate and dream about it afterwards, ‘The kinds of suicide related stories I see… it’s actually very horrifying and very sad… it influences me a lot. I get dreams about it*’* (Female #1). Some even described feeling afraid and mentally disturbed after exposure to suicide-related news, as though consuming this news could trigger suicidal thoughts, ‘We feel a little bit scared also when seeing this news… the impact on us is fear and mental disturbance*’* (Female #8). Several participants found the news overwhelming, causing them to avoid reading it altogether, ‘I feel it is not needed and I don't watch it deeply… everyone gets affected and it is better to avoid*’* (Female #9). Participants also noted that televised reports had a more intensely negative impact on them than reading newspaper reports, *‘*The emotions are conveyed differently on TV… they report the news in an intense manner [and] when the visuals are telecast, it creates an impact*’* (Male #5).

Importantly, a few participants (n=2) who reported recent suicidal thoughts (in the past 12 months) identified that the way media covers suicide incidents can be triggering. Such participants could find the media reports ‘very daunting… and very triggering… if I was not in a somewhat sensible state, I would have actually done something to myself*’* (Female #6). This was particularly enhanced during times when a suicide was getting particularly high-profile repetitive coverage ‘as if nothing else happened in the world, only that one [suicide] happened’ (Female #6). This was also an issue with sensational coverage of suicides on late-night reality shows:

There are some reality shows that get telecast at 10.30pm and 11pm. They select an incident and telecast it with the photos of the victim, and they’ll explain what happened… The content they use is someone’s life and they make it very horrifying to watch. That being telecast at night, I can’t even sleep peacefully… I always avoid… I am myself having some mental health issues, I don’t want to get influenced by watching these (Female #1)

Portrayals of suicidal behaviour trigger the risk of influencing others’ suicidal behaviours, potentially even promoting the idea of suicide as an option and reinforcing that suicide is a solution to their struggles. ‘I do feel that many people might get ideas reading that…. some kids feel like they are prone to attempting it themselves*’* (Female #7) and ‘if someone with mental health issues reads it, they may get ideas about [suicide]*’* (Female #1). Several participants expressed concerns about media coverage of the National Eligibility cum Entrance Test (NEET), a nationwide medical entrance exam that is highly politicised in Tamil Nadu due to its links with debates on social justice and equitable access to education. Participants noted that the media tends to report every suicide linked to the NEET exam failure, and that this ‘news [that] triggers the students*’* (Male #7). In the media:

It has become a habit seeing the list of students clearing the NEET exam on the top row [of the newspaper] and the following row containing the news of the students who committed or attempted suicide for not clearing NEET. Their mind set is just to become a doctor and in case they do not clear the NEET exam then they think the only alternative is suicide… [The reader] will see suicide as a solution (Male #7).

Participants also felt that the media content was often insensitive to the potential impacts on victims and their families. *‘*It will affect their family too… they will highlight the area the person lived in, where they worked, they collect some information from the family*’* (Female #9) and ‘they don’t care about the victim’s family or the victim who committed suicide*’* (Male #3).

#### Missed opportunities to educate the public on how to cope

Participants noted that most suicide reports they encountered lacked information on helplines or guidance on coping with suicidal thoughts. Most participants said they ‘don’t remember seeing helpline numbers’ (Female #7) in media coverage of suicide. Those who did recall seeing them said, ‘you can show the helpline numbers in a prominent way, so people will notice it*’* (Female #4). A prominent belief was that media did not provide coverage on how to cope with hardship and suicidal thoughts. *‘*There is no mention of how to cope with the situation*’* (Female #3) and *‘*they will only blame the person who committed and will telecast it as news but do not provide any solution for suicides… like how it could have been solved*’* (Female #4). Referring to media coverage of a prominent celebrity suicide, ‘it was glorified… no one spoke about why it happened and how it could have been avoided’ (Male #1).

Participants noted the need to *‘*create awareness*’* (Female #4) about how to prevent suicide, including by talking about alternatives to suicide. It was felt that by providing examples of how people have coped with their own suicidal thoughts the audience will learn that they can ‘overcome these thoughts and move on in their life’ (Female #10).

### Exposure to purpose-designed media content on suicide with protective reporting features

Participants also shared their experiences and perspectives in relation to the purpose-designed media articles on suicide that they were exposed to as part of the experiment. Their responses have been presented across three sub-themes.

#### Hope and recovery

Participants frequently shared that the purpose-designed media articles focused on stories of hope and the possibility of recovery. They also shared how this was in sharp contrast to suicide-related media they had previously encountered:

These articles are fully positive. They are about victims coming out of suicidal thoughts. But such news doesn’t come in newspapers… I have not seen even a small paragraph that is printed and reported in a positive way in the newspapers…… I have only read articles of people committing suicide for various reasons… I have not read articles which talk about people fighting with suicidal thoughts and coming out of it (Male #9)

Participants also identified that it was helpful to see articles providing examples of real people who had overcome suicidal thoughts:

The ending of that story was very good. (Rather than ending her life) she got emotionally connected with her sister and mother. That was the reason which made her decide to continue living instead of ending it. Then she started a beauty parlour and got married and lived happily. So, that was a good positive ending. That was good to read. (Male #8).

Here, the participant reflects on the hopeful ending of the story, in which the protagonist secures a job and continues with their life. They noted that this was uncommon in the way such stories are typically portrayed.

Participants felt that typically ‘media only talk about the reasons for the incident, the solution is never told*’* [whereas] i*n these* articles… the solution is explained*’* (Male #2). They noted that a similar issue exists on social media, where only certain perspectives are shared—‘the suicide had happened. But they do not talk about a person who has moved on from that thought*’* (Female #10). In contrast, these purpose-designed media articles showed ‘how they came out of [suicidal thoughts]’ (Female #7), and that ‘it was very solution oriented [rather] than problem oriented… it felt more like hope… I feel there is help and hope*’* (Male #1). For many participants, it was the first time they had seen these kinds of stories and messages about the possibility of recovery from suicidal thoughts, ‘only after reading this article I understand people can come out of this thought and can live their life. (Female #10). Participants were positive about the potential for these stories to have a protective effect on the audience, ‘it’ll help a lot of youth and everyone actually*’* (Male #1):

By publishing positive news and articles there are so many chances for the people to change in a positive way. They’ll get some hope and they’ll start to think that there are so many other reasons to live life. Through positive news, they’ll start to see their past experiences as a lesson and they can also decide to start a new life from that point (Male #8, 21 years).

#### Sparking curiosity, improving knowledge, busting myths

The purpose-designed media articles with protective features sparked a lot of curiosity and interest in the participants. Some participants also reported an improved understanding of suicidal behaviours. For instance, some participants were unaware of the extent of suicide-related deaths, ‘it was very surprising for me …on our way back home we discussed about this study… [and how] only then we came to know that so many people are affected because of suicide’ (Male #9). They advocated for exposure to awareness related content:

The more a person gains knowledge, it is better… there are people around with very minimal knowledge [about suicidal behaviours]. So, if they are educated, they’ll have more knowledge about it. There are high chances, that might lead them to take matured decisions. (Male #8)

Several participants expressed a desire to learn more about suicidal behaviours. Some indicated that this exposure prompted them to look for more content online to deepen their understanding and/or have conversations with their family and friends to share their knowledge, ‘I want to spread this message as much as possible. That’s what I felt…I discussed about it with a few friends*…* it helps in creating awareness about such mental health issues’ (Male #1).

The purpose-designed media articles also served an important function of busting myths. A commonly discussed myth that was the belief that talking about suicide might lead someone to consider it:

I was thinking that by talking about suicide, I might induce suicidal thoughts… Before reading the articles here, I had a very wrong understanding about suicide and suicidal thoughts. But reading the articles was really very helpful for me… (Male #3)

…after reading the articles I understood that we ask others [if we can help] because of the affection we have for them. And in no way it would induce suicidal thoughts in them. (Male #3)

Other participants feared that suicidal thoughts could be contagious but learnt that ‘by speaking to them we are not going to commit or think the same*’* (Female #8). Participants reflected on a range of other learnings, ‘I thought that only mental illness can lead to suicide. actually, it could happen to anyone*’* (Male #6) and that ‘there was a myth that [if you have suicidal thoughts] you’ll always have such suicidal thoughts, and they’ll be full of negativity. But that is not the case.’ (Male #4). Some had also learnt that people with suicidal thoughts can change their minds:

there is a general thought that the minds of the people who have decided to commit suicide cannot be changed at all, by any means. But that is wrong, if someone talks to them, their mindset and their thought to commit suicide can be definitely changed… Only if we talk to them, they’ll understand that there is someone to support and talk to them (Male #3)

Some participants also articulated that the content in these articles adopted a ‘more accepting’ (Female #1) tone towards people who have engaged in suicidal behaviours and the support they need, *‘*without all the glorification*’* (Female #1). This tone seemed to have an impact on participant perspectives, challenging the stigma that people who experience suicidal behaviours are selfish and should be avoided:

What I thought first was they don't have the courage to live, and they are very selfish… so they are doing this. We are scared to have friendship with the person who is having suicidal thoughts. But after reading the article I understood my point of view is totally wrong… I felt they are not coward and they are not selfish… (Female #8)

#### Sources of support

Participants noted that the protective media content they had been exposed to increased their awareness of available sources of support for people experiencing suicidal distress, as well as ongoing programmes aimed at preventing suicide. After reading the articles, ‘I understood that some kind of help is necessary*’* for people with suicidal thoughts (Female #8) and ‘I also learnt, by giving support to such people, we can [help] them to come out of it*’* (Male #3). A key message received was that ‘the content told [people] to not commit suicide by telling [them] there are people here to help…’ (Female #1). Participants also reported learning about the ‘National suicide prevention strategy which will be implemented by government, so there is a prevention strategy*’* (Male #2) and services like the ‘[suicide help centre] which can help people in suicide prevention*’* (Male #9). Learning about the sources of support also led participants to feel that if they were to experience suicidal thoughts in the future, they would know what steps to undertake:

I read about counselling, reducing the sale of pesticides would reduce the suicide count… Getting help from the doctors who talk to the affected people to help them out… I basically have hope [now] that I won’t commit suicide. And even if I get a [suicidal] thought, I now know what to do, that is the main idea that I got from this study (Female #2)

If I have some problem in future, or I feel hopeless and stressful, I will definitely consult a counsellor because I believe they’ll see my problem in a different perspective which might provide a solution (Male #4).

## Discussion

This study provides new insights into how young adults in India perceive suicide coverage in the media, and how they respond to purpose-designed protective content. Participants described the dominant media reporting style as sensational, disturbing and harmful, and based on simplistic assumptions. On the other hand, participants reacted positively to protective content that emphasised hope, recovery and support. These findings add audience’s perspective to a body of literature dominated by analyses of media content and interviews with journalists, and underscore the value of protective reporting strategies in an LMIC setting.

While there is substantial research on the impact of media on suicidality in the last few decades,^14^ audience experiences of media reporting are underexplored. In a qualitative study on experiences of suicide bereavement in India, participants highlighted how language and media sensationalise suicide, reinforcing stereotypes, intensifying grief and restricting access to support.^37^ The study offered insights into how bereaved individuals perceive and experience media reporting of suicides. However, this study is among the few that closely investigates how general audiences experience media reporting, offering a distinctive contribution through its focus on young adults.

Looking into audience experiences is important because media reporting of suicides has been referred to as a double-edged sword,^22^ highlighting its potential for multiple contrasting consequences.^14^ On the one hand, the *Werther effect*^38^ describes how imitation of suicides can be prompted by media coverage. The risk is particularly heightened when reports are sensationalised, such as through front-page placements, graphic descriptions of methods, the use of pictures and bold headlines. This can be particularly harmful to young people, especially those who are depressed and vulnerable.^39 40^ On the other hand, not all reporting on suicides is associated with harmful effects.^9 41^ Portrayals of stories of hope and healing have been found to have protective influences on suicidal behaviours (the *Papageno effect*; 9, 43).

These experiences were reflected in our participant narratives. Given the lack of awareness of and non-adherence to reporting guidelines in India,^20 21 42^ it is unsurprising that the participants had unpleasant responses to general media reporting on suicides. While some were able to dismiss these reports as attention-seeking tactics, others expressed feelings of discomfort and fear when reading such content. This is significant as it challenges media professionals’ understanding that such news is inherently *engaging* for audiences.^27^ Young adults in the study found it anything but engaging, with some intentionally avoiding the news because it was too overwhelming. Their narratives suggested that negative reporting of suicides could be triggering, particularly for those in distress, while failing to encourage help-seeking and potentially reinforcing stigma. This may reflect a missed opportunity to foster meaningful engagement on sensitive yet vital issues such as suicide. These insights are crucial for bridging the gap in media professionals’ understanding of how their reporting is received by its intended audiences.

Interestingly, participants in the study reported feeling surprised, sometimes even shocked, that positive, hopeful content existed, considering its sharp contrast to what they typically encounter in mainstream media. These reactions echo findings from other countries, where exposure to educative content was reported to be rare.^43^ This scarcity is unsurprising as suicide reporting has traditionally focused on dissemination of stories of death rather than narratives of hope. Participants described usually receiving a *one-sided* account that overlooks themes of resilience and recovery beyond the suicidal act. In this context, the protective content served to challenge misconceptions, enhance knowledge and promote help-seeking. Notably, these findings highlight a significant difference between what media professionals and young audiences view as *newsworthy*, suggesting that such stories can be both engaging and impactful, contrary to media professionals’ concerns that they may be *boring* or *ineffective*.^27^

Two notable RCTs have examined the effects of educative suicide prevention content in news articles^43^ and on websites.^44^ Educative content featuring suicide prevention experts, across websites and news articles, improved suicide prevention knowledge and reduced suicidal ideation, particularly in participants with greater vulnerability at baseline.^43 44^ Notably, these effects appear to have stemmed from shifts in participants’ perceptions of their capacity to cope constructively with suicidal ideation and a reinforcement of their survival beliefs.^44^ In general, mechanisms behind both Papageno and Werther effects are insufficiently explored^45^ but a few theories have been proposed.^46^ For example, the *social learning* theory asserts that behaviours are learnt through observation.^47^ Media exerts a powerful influence on people’s behaviours, stimulating both suicidal as well as coping and help-seeking behaviours. In relation to *social identification processes*, it has been found that audiences may strongly relate to main protagonists featured in stories of hope and recovery. This may accordingly influence a reduction in suicidal thoughts.^48 49^ The content used in this study did not feature protagonists or narratives, nor did it investigate mechanisms through which protective influences occur. Future research, particularly in low-and middle-income contexts, could explore which elements of a message exert a protective influence and the underlying mechanisms driving these influences.

### Implications

To our knowledge, this is the first qualitative exploration of youth perspectives on media and suicide in India. Participants’ accounts confirmed existing research and guidelines (eg, WHO) affirming that sensationalistic, graphic, detail-heavy suicide reporting can be emotionally disturbing, triggering and may contribute to contagion. They also affirmed that exposure to hope-oriented educative stories sparked curiosity, boosted their sense of hope, corrected myths, increased awareness about supports available and encouraged help seeking. This seemed to be in sharp contrast to what journalists understand as *newsworthy* or *exciting* news.^27^ Findings from the study suggest that young adults hold clear views about what is helpful and harmful in the media. These audience insights can be a powerful resource for co-designing constructive media content.

The findings may also inform media professionals about the impact of their media reports. Despite the adoption of media guidelines in India and some initiatives to guide journalists towards improved coverage of suicide, there has been minimal change in actual reporting practices.^50^ Our study highlights the potent stories captured through audience research, which may be able to play a crucial role in efforts to engage with media professionals. Up to now there has been limited integration of audience experiences in media and suicide research. Understanding how audiences perceive and respond to news content may address some of the aforementioned attitudinal barriers to implementation reported by media professionals. Audience perspectives can play a pivotal role in challenging assumptions about the real-world impact of reporting and bring to life the ethical challenges faced by journalists when covering suicide-related content. This could help inform potential implementation considerations. These perspectives may also be used to inform the co-design of workshops for media professionals and other stakeholders.

### Strengths and limitations

The use of qualitative methods enabled a detailed exploration of participants’ experiences, offering insights into how young people perceive suicide reporting in the media. With 20 interviews, we achieved rich data and thematic saturation, generating descriptions that captured the meanings and interpretations drawn by youth. We also aligned the study with a trial where participants were exposed to purpose-designed protective content. This approach provided a valuable opportunity to capture meaningful insights into how protective stories spark curiosity, challenge myths and encourage help-seeking.

Some important limitations need to be acknowledged. Our participants were all urban, Tamil-speaking young adults living in Chennai city, which could limit the transferability of our findings. Due to resource and time constraints, the analysis focused on the aggregate range of participant experiences rather than conducting a detailed comparison between the two intervention groups. As a result, potential differences in how participants responded to the educative content vs the lived-experience narratives could not be systematically examined. Perspectives from rural youth, or from young people in other linguistic and cultural settings may differ in important ways. Given India’s cultural diversity, the experiences of this relatively homogenous group should not be assumed to reflect the views of young adults nationwide. Future research that engages youth from a broader range of regions and backgrounds will be essential to expand the evidence base.

## Conclusion

This study is among the first to explore young adults’ experiences of media reporting of suicide in a low- and middle-income context. Young adults’ narratives revealed what they consider to be helpful and harmful in suicide-related news, highlighting that the solution is not to silence media coverage but to use it as an important opportunity to raise awareness, foster action and encourage help-seeking. Importantly, these findings provide actionable feedback for designing training and capacity-building initiatives for media professionals, policymakers and other stakeholders. Integrating audience perspectives into the large-scale adoption of media guidelines will be critical for ensuring that reporting is both safe and meaningful across diverse contexts.

## Supplementary material

10.1136/bmjopen-2025-113009 online supplemental file 1

10.1136/bmjopen-2025-113009 online supplemental file 2

## Data Availability

All data relevant to the study are included in the article or uploaded as supplementary information.
