# Supplementary material for: Young adult perspectives on media content related to suicide in South India: a qualitative study
Source: BMJ Open. 2026 May 27;16(5):e113009. doi: 10.1136/bmjopen-2025-113009 (PMC13218101; doi:10.1136/bmjopen-2025-113009)
Supplement: online supplemental file 1 [file bmjopen-16-5-s001.docx]

**Supplementary material 1**

**Consolidated criteria for REporting Qualitative studies (COREQ) checklist**

| **Domain 1: Research team and reflexivity** | |
| --- | --- |
| ***Personal Characteristics*** | |
| **Interviewer/facilitator**  (Which authors conducted the interview process) | SK and RM  Data collection sub-section, page 9 |
| **Credentials**  (What were the researcher’s credentials?) | GA (Lead investigator, PhD International Health); SK (PhD Psychology); RM (PhD Psychology); SKr (PhD Public health); LV (PhD, MD Psychiatry)  Data collection sub-section, page 9 |
| **Occupation**  (What was their occupation at the time of the study?) | GA (Associate Professor); SK (social scientist/researcher); RM (psychologist); SKr (Research Fellow); LV (Psychiatrist)  Data collection sub-section, page 9 |
| **Gender** | GA (Male); SK (Female); RM (Female); SKr (Female); LV (Female)  Data collection sub-section, page 9 |
| **Experience and training**  (What experience or training did the researcher have?) | GA: Senior researcher and consultancies in Australia and in low and middle-income countries (LMICs) over the past 10+ years with an interest in population mental health, suicide prevention.  SK: Trained in quantitative and qualitative research methods, consultancies on using both qualitative and quantitative research methods in national and international multi-centric health projects.  RM: Trained in quantitative and qualitative research methods, psychologist and social scientist, consultancies on using both qualitative and quantitative research methods in national and international multi-centric health projects.  SKr: Trained in qualitative research; mental health and suicide prevention project implementation experience in low- and middle-income countries (LMIC)  LV: Leading suicide researcher in LMICs with over 25+ years of experience.  Data collection sub-section, page 9 |
| ***Relationship with participants*** | |
| **Relationship established**  (Was a relationship established prior to study commencement?) | Data collection sub-section, page 9  This study was nested within a larger randomised controlled trial. Participants for the qualitative study were drawn from this study. approached. Following completion of their participation in the trial, a purposive sub-sample of 20 youth (10 males and 10 females) consented to participate in semi-structured qualitative interviews, with a mix of participants from both intervention groups.  Before the start of the interview process, the interviewers introduced themselves. Participants were given an opportunity to understand what the interview process entails. Interviewers (SK and RM) had no prior relationship with the participants. |
| **Participant knowledge of the interviewer**  (What did the participants know about the researcher? e.g. personal goals, reasons for doing the research) | Data collection sub-section, page 9  Interviewers shared their name, qualifications, and professional experience. |
| **Interviewer characteristics**  (What characteristics were reported about the interviewer/facilitator? e.g. Bias, assumptions, reasons and interests in the research topic) | Data collection sub-section, page 9  Interviewers reported their interest in the research topic. For example – interviewers’ interest in how suicide related news is perceived by audiences. |
| **Domain 2: Study Design** | |
| ***Theoretical framework*** | |
| **Methodological orientation and Theory**  (What methodological orientation was stated to underpin the study?) | Details related to the design of the study have been mentioned in the Study design sub-section (page 7). |
| ***Participant selection*** | |
| **Sampling**  (How were the participants selected?) | Purposive sampling technique  Details related to the sample and sampling technique are mentioned in the Sample sub-section (page 8) |
| **Method of approach**  (How were participants approached? e.g. face-to-face, telephone, mail, email) | Face to face  Data collection sub-section, page 9 |
| **Sample size**  (How many participants were in the study?) | 20 young adults: 10 males and 10 females  Data collection sub-section, page 10 |
| **Non-participation**  (How many people refused to participate or dropped out? Reasons?) | RCT participants were asked about their interest in a follow-up qualitative study, and those who agreed were subsequently contacted. Non-response was not noted.  Sample sub-section, page 8 |
| ***Setting*** | |
| **Setting of data collection**  (Where was the data collected? e.g. home, clinic, workplace) | Workplace – in a private room/setting  Data collection sub-section, page 9 |
| **Presence of non-participants**  (Was anyone else present besides the participants and researchers?) | No one else was present besides the participants and interviewers.  Data collection sub-section, page 9 |
| **Description of sample**  (What are the important characteristics of the sample? e.g. demographic data, date) | The sample comprised an equal number of male (n=10; 50%) and female (n=10; 50%) aged between 18 and 29 (see Table 1). Most participants were undergraduate or postgraduate students specialising in a range of disciplines such as science, commerce, fashion, creative arts and practice. Of these, eight participants were employed in various roles, including positions in an IT department, as a manager in a private hospital, as an athlete, in sales, and as a photographer. Most participants (n=18, 90%) reported not having recent suicidal thoughts (in the past 12 months). Participants were also asked about their recent exposure to suicide related news. While one participant could not recall any such exposure, most participants (n=8, 40%) indicated that they had not been exposed to such news at all in the past two weeks. A few mentioned that they had been exposed to suicide related news once (n=5, 25%) or twice (n=6, 30%) in the past two weeks.  Results section, page 14, More details in Table 1. |
| ***Data collection*** | |
| **Interview guide**  (Were questions, prompts, guides provided by the authors? Was it pilot tested?) | Details related to the interview guide are discussed in the Data collection sub-section, page 10. The interview guide was not pilot tested. |
| **Repeat interviews**  (Were repeat interviews carried out? If yes, how many?) | No repeat interviews were carried out.  Data collection sub-section, page 10. |
| **Audio/visual recording**  (Did the research use audio or visual recording to collect the data?) | Audio recording, following written consent from participants.  Data collection sub-section, page 9. |
| **Field notes**  (Were field notes made during and/or after the interview or focus group?) | Field notes taken after each interview. Analytic memos were maintained throughout the coding process.  Data analysis sub-section page 11 |
| **Duration**  (What was the duration of the interviews or focus group?) | 45-60 minutes.  Data collection sub-section, page 9. |
| **Data saturation**  (Was data saturation discussed?) | Data saturation discussed with team members (GA, LV)  Data collection sub-section, page 10. |
| **Transcripts returned**  (Were transcripts returned to participants for comment and/or correction?) | Transcripts were not returned to participants for comments and/or correction.  Data analysis sub-section, page 11 |
| **Domain 3: Analysis and findings** | |
| ***Data analysis*** | |
| **Number of data coders**  (How many data coders coded the data?) | Two coders - SK and RM with supervision and support from GA and LV.  Data analysis sub-section, page 11 |
| **Description of coding tree**  (Did authors provide a description of the coding tree?) | A deductive analytic approach was followed to corroborate and complement findings of the trial. The code list was derived from the topics discussed in the interview guide.  Data analysis sub-section, page 11 |
| **Derivation of themes**  (Were themes identified in advance or derived from the data?) | Themes were identified through the process of coding but were largely based on the topics discussed in the interview guide.  Data analysis sub-section, page 11 |
| **Software**  (What software, if applicable, was used to manage the data?) | NVivo  Data analysis sub-section, page 11 |
| **Participant checking**  (Did participants provide feedback on the findings?) | No, participants did not provide feedback on the findings.  Data analysis sub-section, page 11 |
| ***Reporting*** | |
| **Quotations presented**  (Were participant quotations presented to illustrate the themes / findings? Was each quotation identified? e.g. participant number) | Quotations presented with deidentified participant numbers.  Results section (page 13-22) |
| **Data and findings consistent**  (Was there consistency between the data presented and the findings?) | There seems to be consistency between data and findings**.**  Results section (page 13-22) |
| **Clarity of major themes**  (Were major themes clearly presented in the findings?) | Definitions of main and sub themes provided in the Results section (page 13-14). |
| **Clarity of minor themes**  (Were minor themes clearly presented in the findings?) | Definitions of main and sub themes provided in the Results section.  Results section (page 13-22) |
