# Supplementary material for: Young adult perspectives on media content related to suicide in South India: a qualitative study
Source: BMJ Open. 2026 May 27;16(5):e113009. doi: 10.1136/bmjopen-2025-113009 (PMC13218101; doi:10.1136/bmjopen-2025-113009)
Supplement: online supplemental file 2 [file bmjopen-16-5-s002.docx]

**Supplementary material 3**

**Interview guide**

# Our approach

Objective:

The study objective is to examine participant perceptions of the attitudinal, behavioural and emotional impacts of the print media articles on themselves and their peers. We also ask participants about their experience of exposure to suicide content in the media, and how the print media articles we used in the experiment are different to the kinds of content they usually see.

Participants: We will conduct semi-structured qualitative interviews with 20 participants (10 from each of the two intervention groups; even gender split, even age split) to investigate responses to the suicide prevention messaging in the newspaper articles.

Data collection:

- Participants will undertake an audio-recorded 45-minute semi-structure qualitative

interview with the India-based senior researcher, after having been given time to re-read the articles they were exposed to in the experiment.

- A discussion guide will be designed to capture information on participant perceptions of the attitudinal, behavioural and emotional impacts of the print media articles on themselves and their peers.
- Participants will also be encouraged to talk freely about other responses they have in

relation to the articles.

Data analysis:

- Audio recordings will be transcribed and translated by the India-based senior

researcher.

- Transcripts will be analysed by Greg, Rani & Shuba using a mix of deductive

and inductive thematic analysis, supported by NVivo.

Rigour:

To ensure a high level of rigour, we will implement a number of recommended strategies. For example:

- the discussion guide will be tested at pilot interviews
- there will be regular debriefing meetings aided by the use of a reflexive journal kept by the interviewer

# Discussion guide

## Before starting:

## Give the participant time to re-read the three articles they had been given during the study

Participant ID:_____________________

**We’d like to start by asking you a little about the kinds of suicide-related stories that you typically see in the media**

1. Where have you typically seen media content related to suicide?

*Prompts:*

- - *Newspapers, news channels, tv series, movies, social media? It could be fiction or non-fiction.*

1. Please describe the nature of the media content that you typically see?

*Prompts:*

- - - *Graphic depictions of suicide?*
    - *Information about rising suicide rates? Information about suicide prevention helplines or services?*
    - *Discussion of groups at risk, like students and farmers?*
    - *Personal disclosures of suicidal thoughts/experiences/bereavement?*

1. How do you typically feel when seeing this kind of media content?

*Prompts:*

- *felt nothing, sad, hopeful/hopeless, angry, overwhelmed or something else?*
- *What are your thoughts when you read or see media content about suicides and about the persons who committed?*

1. Do you think the media content you typically see has any effects on people who are exposed to it? If so, what types of effects?

*Prompts:*

- *What about effects on people who are thinking about suicide?*

**Now we’d like you reflect on the media articles that you saw during this study**

1. Thinking about the media articles that you read as part of our study, do they seem similar or different to the suicide content you have typically seen in the media?

*Prompts:*

- *If different, what specific aspects are different?*
- *If similar, what aspects are similar?*

**The next questions is for those people who were given media articles that contained stories of people who had found a way to cope during a period of having suicidal thoughts**

1. Had you previously seen any media content where people are discussing the ways they have coped during a period of having suicidal thoughts?

*Prompts:*

- *Where did you see this type of content before? Were they regular people or celebrities who were sharing their stories?*

1. Do you think it would be helpful to see more stories of how people cope with suicidal thoughts?

*Prompts:*

- *If so, why?*
- *If not, why not?*

1. What kind of media content would you like to see that looks at stories of how people are coping with suicidal thoughts?

*Prompts:*

- *Specific situations people experience? Specific demographic groups?*
- *What media format/source, etc?*

1. Do you have any other suggestions on how we can improve these media articles and make them more interesting/appealing?

**We’d like to ask you a few questions about whether reading these media articles had any impact on you.**

1. How did you feel emotionally after reading these media articles?

*Prompts:*

- *Felt nothing, sad, hopeful or hopeless, angry, overwhelmed etc?*
  - *If a negative feeling, ask what about the media articles made them feel that way? Also, follow-up by asking if they were still glad they read the media articles and why?*
  - *If a positive feeling, ask what about the media articles made them feel that way?*
  - *Have you had a previous experience of feeling suicidal, or a family/friend? Did the articles cause you to reflect on those experiences*

1. Did the media articles in any way change your attitudes towards other people who experience suicidal thoughts?

*Prompts:*

- *In what way? Negatively or positively?*

1. Did the media articles in any way change how you feel about supporting someone who is experiencing suicidal thoughts?

*Prompts:*

- *In what way?*

1. Did the media articles stimulate you to find out more information about suicide?

*Prompts:*

- *If yes, what kinds of information?*
- *What sources of information did you try? (friends, internet, etc)*

**Finally, we’d like to ask whether you think media articles like these would have any positive or negative impacts on people in the community**

1. Do you think media articles like these will have a positive or negative impact on people who read them?

*Prompts:*

- *How would these kinds of media articles have an impact?*
- *What types of impacts? (providing help to those in crisis, feeling hopeful for being able to prevent suicide, change attitudes, stigma etc)*

1. What effects would these articles have on someone who was thinking about suicide?

*Prompts:*

- *How would these kinds of media articles have an impact?*
- *What types of impacts? (seeking help, feeling hopeful for the future, awareness about suicide prevention helplines etc)*

### Interview complete:

### Thank the participant

### Implement debriefing protocol
