# Supplementary material for: Young adult perspectives on media content related to suicide in South India: a qualitative study
Source: BMJ Open. 2026 May 27;16(5):e113009. doi: 10.1136/bmjopen-2025-113009 (PMC13218101; doi:10.1136/bmjopen-2025-113009)
Supplement: Supplementary file 3 [file bmjopen-2025-113009.reviewer_comments.pdf]

PEER REVIEW HISTORY

BMJ Open publishes all reviews undertaken for accepted manuscripts. Reviewers are asked to complete a checklist review form and are provided with free text boxes to elaborate on their assessment. These free text comments are reproduced below.

ARTICLE DETAILS

Title (Provisional)

Young adult experiences of suicide-related media content in South India: a qualitative study

Authors

Armstrong, Gregory; Kumar, Shuba; Mohanraj, Rani; Krishnamoorthy, Sadhvi; Vijayakumar , Lakshmi

---

VERSION 1 - REVIEW

**Reviewer**	**1**
**Name**	**Groves, Samantha**
**Affiliation**	**University of Oxford Department of Psychiatry**
**Date**	**13-Nov-2025**
**COI**	**None**

Research Summary and Overall Impression

Many thanks for the opportunity to review this article. This is an important and interesting study given the high incidence of suicide in India, and the growing field of research examining the potential for a 'Papageno effect' related to responsible media coverage of suicide. The study involves a qualitative study nested within a larger trial examining the impact of hope-centred messages surrounding suicide on young adults. I hope the below comments are useful.

General Comment: An additional round of proofreading to check for minor errors, sentence clarity, and word choices would be useful. For example, on page 8, line 28, there are two full stops, and at the end of the reflexivity paragraph, there appears to be one missing.

Background:

- *Page 5, line 34:* I found this sentence a bit difficult to read- rewording to something like the following may improve readability: *'However, less is known about the potential protective effects of responsible media reporting of suicide"*
- *Page 5, line 49:* the first sentence would benefit from specifically stating that the guidelines are related to reporting of suicide e.g. "In 2023, the World Health Organization (WHO) and the International Association for Suicide Prevention (IASP) partnered to develop international guidelines for media professionals reporting stories about suicide"

- *Page 5, line 59:* it would be helpful to inform the reader about the 2019 guidance before mentioning its adoption by the press council of India- at the moment it's slightly confusing to learn about the 2023 guidance (WHO + IASP) but then mention the adoption of different guidance by the press council.
- *Page 6, lines 28-30:* it would be interesting to briefly outline measured trial outcomes (e.g., what types of effects related to exposure are being measured by the trial?
- *Throughout*: At the end of the introduction, I learned about the trial sample of young adults. I was wondered whether throughout the introduction a clear rationale for this sample could be weaved in e.g., is there evidence related to the impact of exposure to media coverage of suicide among young adults to highlight?

Methods:

- As this study is nested within a trial, I think it would be beneficial to start this section with a brief subheading and summary of the wider trial. This could allow the wider context of the study to be understood, (e.g., the recruitment of participants into the RCT, description of the trial arms etc). The remainder of the methods could then focus specifically on the qualitative component.
- *Page 6, line 53:* the RCT is mentioned within the introduction so the abbreviation could be introduced the first time the term is used.
- *Page 7, line 22*: Some extra detail could be added surrounding how participants were drawn from the RCT to the qualitative study. Later it is stated that a purposive sample was used, so it would be helpful to state what characteristics were targeted.
- *Page 8, line 5*: a small point, but it is important to acknowledge the researcher's active role within generation of themes during thematic analysis, i.e. themes don't emerge from the data, we instead work to construct them, see Braun and Clarke: https://doi.org/10.1080/2159676X.2019.1628806
- *Page 8, line 10:* I'd be interested to know how long the gap between the trial intervention and the qualitative study was? e.g., was it the same day, a week later etc.
- *Page 8, line 15:* it would be helpful to understand how the content of the topic guide was developed e.g., were questions based on a theory/ framework/ relevant literature/ or outcomes of interest measured within the wider trial? Optionally, you could add the topic guide as a supplementary file.
- *Page 8, line 46:* it appears (as the acronym is introduced here) that this is the first time the semi-structured nature of the interview is discussed, it would be useful to introduce this within the design section of the methods and provide a rationale why semi-structured interviews were chosen.
- *Page 8*: it would be helpful to provide a brief paragraph explaining steps taken to enhance the rigour and trustworthiness of the study. For example, Lincoln and Guba's (1985) criteria of credibility, transferability, dependability, and confirmability.
- *Page 9, line 18:* should it be male and females?
- Page 9, line 30: the word 'most' could be changed if referring to 40% of the sample.
- *Page 9:* within the study design section of the methods, it would be useful to explain how to descriptive data of the participants were gathered e.g., did they fill in a form before/after the interview?

Results

The two presented themes closely reflect the study aims (i.e., experiences and impact of exposure in everyday life, and experiences and perceived impact of exposure to the intervention arms). The close alignment between the study aims and the themes suggest that analysis was quite deductive, as these aims were established prior to data collection and analysis. I think this structure makes sense

as it will complement the findings of the trial. However, I think some more clarity as to the weighting of deductive and inductive coding would be valuable within the data analysis section of the methods.

I would also be interested to hear whether there were any differences/similarities between the views of participants within the two intervention groups (e.g., were the lived experience accounts specifically discussed?).

Additionally, were there any contradicting views shared by participants which may be important to consider? E.g., were any viewpoints related to implementation shared i.e., being unsure whether media professionals would adopt such stories?

- *General*: In a couple of places, the quotes are slightly difficult to read or not or the sentence structure could more clearly state these are the views shared by participants, some proofreading may be helpful just to enhance readability:
    - E.g., the following sentence could be reworded to "Some participants shared that they felt portrayals of suicidal behaviour could trigger the risk of influencing others' suicidal behaviours…" *(Page 14, line 34)*
    - e.g., taking away the I from this quote to help sentence flow: "After reading the articles one participant shared that they '~~I~~ understood that some kind of help is necessary' for people with suicidal thoughts' (Female #8, 22 years)" *(Page 18, line 37)*
- Page 11, line 55: in the text it states seven subthemes, but I can only see six within the table/text.
- Page 12, line 22: I wondered whether the paragraph surrounding potential impact of coverage on family may be better suited to the second subtheme effects of exposure?
- Page 15, line 13: this paragraph is quite difficult to read due to having quite a few quotes. Perhaps splitting the paragraph with a bit more commentary surrounding it may help readability?
- Page 17, line 14: appears that a study code for the participant has been used instead of the descriptor
- I found the reflection of the individuals with lived experience of suicidal thoughts interesting in the first theme- I was wondering about this group's views of the purpose designed articles.

<u>Discussion</u>

- Page 19, line 49: in the following sentence it would be helpful to explain what effects were measured e.g., knowledge/ suicide ideation. "Articles featuring suicide prevention experts – whether they had lived experience, resulted in comparable on … [38]"

VERSION 1 - AUTHOR RESPONSE

Reviewer 1:

Research Summary and Overall Impression

Many thanks for the opportunity to review this article. This is an important and interesting study given the high incidence of suicide in India, and the growing field of research examining the potential for a 'Papageno effect' related to responsible media coverage of suicide. The study involves a qualitative study nested within a larger trial examining the impact of hope-centred messages surrounding suicide on young adults. I hope the below comments are useful.

General Comment: An additional round of proofreading to check for minor errors, sentence clarity, and word choices would be useful. For example, on page 8, line 28, there are two full stops, and at the end of the reflexivity paragraph, there appears to be one missing.

Response: Thank you. This is noted. The manuscript has been thoroughly proofread and errors such as these have been addressed.

Background:

- Page 5, line 34: I found this sentence a bit difficult to read- rewording to something like the following may improve readability: 'However, less is known about the potential protective effects of responsible media reporting of suicide"
  Response: Thank you. The specific sentence mentioned here could not be found. The sentence that most closely relates to this one is on page 5, paragraph 3 – "However, there has been less research into the potential to generate protective effects from media stimuli." This sentence has been rephrased for further clarity.

- Page 5, line 49: the first sentence would benefit from specifically stating that the guidelines are related to reporting of suicide e.g. "In 2023, the World Health Organization (WHO) and the International Association for Suicide Prevention (IASP) partnered to develop international guidelines for media professionals reporting stories about suicide"
  Response: Noted. This has been rephrased in the text (page 5, paragraph 4, line 49).

- Page 5, line 59: it would be helpful to inform the reader about the 2019 guidance before mentioning its adoption by the press council of India- at the moment it's slightly confusing to learn about the 2023 guidance (WHO + IASP) but then mention the adoption of different guidance by the press council.
  Response: Noted. This has been rephrased in the text (page 6, paragraph 4)

- Page 6, lines 28-30: it would be interesting to briefly outline measured trial outcomes (e.g., what types of effects related to exposure are being measured by the trial?
  Response: Noted. Details related to trial outcome measures have been added (paragraph 2, page 6)

- Throughout: At the end of the introduction, I learned about the trial sample of young adults. I was wondered whether throughout the introduction a clear rationale for this sample could be weaved in e.g., is there evidence related to the impact of exposure to media coverage of suicide among young adults to highlight?
  Response: Thank you. The relevance of addressing suicides among young adults in India has been discussed in the opening (page 5) and closing paragraph (page 6) of the Introduction section.

Methods:

- As this study is nested within a trial, I think it would be beneficial to start this section with a brief subheading and summary of the wider trial. This could allow the wider context of the study to be understood, (e.g., the recruitment of participants into the RCT, description of the trial arms etc). The remainder of the methods could then focus specifically on the qualitative component.
  Response: Thank you. This has been added to the introductory paragraph of the Methods section (under Study design sub-section, page 7). Some of this information was mentioned under the Participants (now rephrased as "Sample") sub-section. This has been removed to ensure there are no repetitions.

- Page 6, line 53: the RCT is mentioned within the introduction so the abbreviation could be introduced the first time the term is used.
  Response: Thank you. "RCT" is now used as an acronym in this section.

- Page 7, line 22: Some extra detail could be added surrounding how participants were drawn from the RCT to the qualitative study. Later it is stated that a purposive sample was used, so it would be helpful to state what characteristics were targeted.
  Response: Noted. The first paragraph under the Data collection sub-section, has now been moved to the Sample sub-section, since it was more to do with the sampling process. Specific characteristics such as an equal representation of males and females, from both intervention groups were targeted. This has been clarified in the Sample sub-section, page 8.

- Page 8, line 5: a small point, but it is important to acknowledge the researcher's active role within generation of themes during thematic analysis, i.e. themes don't emerge from the data, we instead work to construct them, see Braun and Clarke: https://doi.org/10.1080/2159676X.2019.1628806
  Response: Thank you. This has been acknowledged under the Data Analysis sub-section (paragraph 1, page 11). The above-mentioned reference has also been cited to substantiate this statement.

- Page 8, line 10: I'd be interested to know how long the gap between the trial intervention and the qualitative study was? e.g., was it the same day, a week later etc.
  Response: Noted. The qualitative interviews were conducted within two tweels of their participation in the trial, on the same day. They were given time to re-read the print media content they had been given as party of the study to refresh their memory. This has been added to the Data collection sub-section (page 9).

- Page 8, line 15: it would be helpful to understand how the content of the topic guide was developed e.g., were questions based on a theory/ framework/ relevant literature/

or outcomes of interest measured within the wider trial? Optionally, you could add the topic guide as a supplementary file.
Response: Noted. This is an important point. The interview guide was developed with specific objectives in mind. We wanted to explore participants' experiences related to suicide related media content prior to the trial. We also wanted to understand their perspectives on the content they were shown in the trial.
In addition, we wanted to explore the impact of the intervention on their own experiences of distress (if any; primary outcome); their suicide prevention knowledge; stigmatising attitudes; and help seeking behaviours (secondary outcomes). To summarise, we wanted to gather more qualitative data on our outcomes of interest. The guide was developed keeping these objectives in mind. This has been clarified in the manuscript under the Data collection sub-section (page 10).
We have now attached the interview guide as supplementary material 2.

- Page 8, line 46: it appears (as the acronym is introduced here) that this is the first time the semistructured nature of the interview is discussed, it would be useful to introduce this within the design section of the methods and provide a rationale why semi-structured interviews were chosen.
  Response: Noted. We have included this in the Study design sub-section (page 7).

- Page 8: it would be helpful to provide a brief paragraph explaining steps taken to enhance the rigour and trustworthiness of the study. For example, Lincoln and Guba's (1985) criteria of credibility, transferability, dependability, and confirmability.
  Response: Thank you. We have added a sub-section (2.6) on Study rigour (page 11 and 12).

- Page 9, line 18: should it be male and females?
  Response: Noted. This has now been addressed.

- Page 9, line 30: the word 'most' could be changed if referring to 40% of the sample.
  Response: Noted. This has been changed.

- Page 9: within the study design section of the methods, it would be useful to explain how to descriptive data of the participants were gathered e.g., did they fill in a form before/after the interview?
  Response: Thank you, this is an important point. Participants were assigned a unique identification number during baseline data collection prior to the trial, when demographic information was also collected. Participants who took part in the qualitative study provided this ID, allowing the research team to retrieve their demographic data without requiring them to repeat this information. We have added this in the Data collection sub-section (page 9).

Results

- The two presented themes closely reflect the study aims (i.e., experiences and impact of exposure in everyday life, and experiences and perceived impact of exposure to the intervention arms). The close alignment between the study aims and the themes suggest that analysis was quite deductive, as these aims were established prior to data collection and analysis. I think this structure makes sense as it will complement the findings of the trial. However, I think some more clarity as to the weighting of deductive and inductive coding would be valuable within the data analysis section of the methods.
  Response: Thank you. Yes, data analysis was guided by the study aims and was established prior to data collection. The qualitative component was designed to complement and help interpret the findings of the trial. However, a combination of deductive and inductive methods for analysis was used. The major themes were determined deductively, from the objectives of the study. In contrast, the codes contributing to the sub-themes were derived inductively during the data coding process, allowing for the inclusion of insights that extended beyond the initial conceptualisation. This has now been clarified throughout in the Data Analysis subsection (page 11).

- I would also be interested to hear whether there were any differences/similarities between the views of participants within the two intervention groups (e.g., were the lived experience accounts specifically discussed?). Additionally, were there any contradicting views shared by participants which may be important to consider? E.g., were any viewpoints related to implementation shared i.e., being unsure whether media professionals would adopt such stories?
  Response: Thank you, this is a very important point. Due to resource and time constraints, our qualitative analysis focused on understanding participants' experiences and perspectives following exposure to the media content. Therefore, we did not systematically examine how perspectives differed between participants exposed to educative content alone and those exposed to content that also included lived experience accounts. While participants across interviews reflected on elements such as messages of hope, help-seeking, and sources of support, the analysis was not designed to attribute these views specifically to each intervention arm. Similarly, although participants occasionally raised broader reflections about media portrayals of suicide, including considerations about how such stories might be presented, the study did not specifically explore implementation-related perspectives (e.g., whether media professionals would adopt such reporting practices) in depth. We have included this as a limitation (page 25) and acknowledge that future research could more explicitly compare participant responses across intervention conditions and explore potential implementation considerations (page 25).

- General: In a couple of places, the quotes are slightly difficult to read or not or the sentence structure could more clearly state these are the views shared by participants, some proofreading may be helpful just to enhance readability: E.g., the following

sentence could be reworded to "Some participants shared that they felt portrayals of suicidal behaviour could trigger the risk of influencing others' suicidal behaviours…" (Page 14, line 34) e.g., taking away the I from this quote to help sentence flow: "After reading the articles one participant shared that they 'I understood that some kind of help is necessary' for people with suicidal thoughts' (Female #8, 22 years)" (Page 18, line 37)
Response: Noted. We have now thoroughly checked all the quotes and proofread them to enhance readability.

- Page 11, line 55: in the text it states seven subthemes, but I can only see six within the table/text.
Response: Noted. This has now been corrected.

- Page 12, line 22: I wondered whether the paragraph surrounding potential impact of coverage on family may be better suited to the second subtheme effects of exposure?
Response: Noted. This has been moved to the second sub-theme on the effects of exposure (page 18)

- Page 15, line 13: this paragraph is quite difficult to read due to having quite a few quotes. Perhaps splitting the paragraph with a bit more commentary surrounding it may help readability?
Response: Thank you. This paragraph has been rephrased for clarity.

- Page 17, line 14: appears that a study code for the participant has been used instead of the descriptor. I found the reflection of the individuals with lived experience of suicidal thoughts interesting in the first theme- I was wondering about this group's views of the purpose designed articles.
Response: Noted. The code has been rectified, and all additional identifiers removed. Given the very small number of participants with lived experience (n = 2), no further reflections on the purpose-designed articles from this group could be identified. We are doing further research specifically targeting people with lived experience to better understand their specific experiences.

Discussion

- Page 19, line 49: in the following sentence it would be helpful to explain what effects were measured e.g., knowledge/ suicide ideation. "Articles featuring suicide prevention experts –whether they had lived experience, resulted in comparable on … [38]"
Response: Noted. This has been rephrased to add details related to the effects measured (page 24).

VERSION 2 - REVIEW

Reviewer 1
Name Groves, Samantha
Affiliation University of Oxford Department of Psychiatry
Date 23-Apr-2026
COI

Thank you for the opportunity to review the resubmission. The authors have addressed all comments. I only have minor comment which could be quickly rectified at the proof stage-

'In 2019, the Press Council of India provided guidance based on the WHO media guidelines for the Indian context'. The WHO guidelines referenced were published 2023. Maybe it just needs to be clarified that the 2019 guidance was based upon a previous set of guidance?

VERSION 2 - AUTHOR RESPONSE

Reviewer: 1
Ms. Samantha Groves, University of Oxford Department of Psychiatry
Comments to the Author:
Thank you for the opportunity to review the resubmission. The authors have addressed all comments. I only have minor comment which could be quickly rectified at the proof stage-

'In 2019, the Press Council of India provided guidance based on the WHO media guidelines for the Indian context'. The WHO guidelines referenced were published 2023. Maybe it just needs to be clarified that the 2019 guidance was based upon a previous set of guidance?

Response: Noted. This has been addressed (page 6).
